# Influence of noise-binding energy interplay on DC-Kerr effect and electro-absorption coefficient of impurity doped quantum dots

**DOI:** 10.1016/j.heliyon.2019.e01832

**Published:** 2019-05-30

**Authors:** Anuja Ghosh, Sk. Md. Arif, Manas Ghosh

**Affiliations:** Department of Chemistry, Physical Chemistry Section, Visva-Bharati University, Santiniketan, Birbhum 731235, West Bengal, India

**Keywords:** Materials science

## Abstract

Present investigation focuses on analyzing the role of *noise-binding energy (BE) interplay* on the *correction factors (CF)* related to *Kerr nonlinearity*, *DC-Kerr effect (DCKE)* and *electro-absorption coefficient (EAC)* of *GaAs* quantum dot (QD) contaminated with impurity under the active presence of *Gaussian white noise*. The dopant impurity is modeled by a Gaussian potential. The noise-BE interplay does not give rise to any new interesting feature in case of CF from that in absence of noise. However, the said interplay prominently influences the DCKE and EAC profiles. This is justified by the emergence of distinct qualitative characteristics in the DCKE and EAC profiles that evidently depend on the mode of introduction of noise.

## 1 Introduction

Last few decades have witnessed unabated research on low-dimensional semiconductor systems (LDSS) e.g. quantum wells (QWLs), quantum wires (QWRs) and quantum dots (QDs). The impetus for the said research has both technological and academic origins. The technological origin being the widespread application of LDSS in the manufacture of microelectronic and optoelectronic devices having very high efficacy such as QD lasers, solar cells, single electron transistors and quantum computers. And, from an academic perspective, study of LDSS physics refreshes many important concepts of quantum mechanics. Studies of LDSS physics earn further delicacy with the introduction of impurity (dopant) which induces a noticeable interaction between the original LDSS confinement potential and the newly arrived impurity potential. As a result, various physical properties of LDSS (electronic, magnetic, optical etc.) are changed from that of an impurity-free condition. The changed values of these properties are quite encouraging from a technological viewpoint. Thus, we come across a plenty of relevant studies on LDSS physics with proper thrust on dopant contributions [1], [2], [3], [4], [5], [6], [7], [8], [9], [10], [11], [12], [13], [14], [15], [16], [17], [18], [19], [20], [21], [22], [23], [24], [25], [26], [27], [28], [29], [30], [31], [32], [33], [34], [35], [36], [37].

*Kerr effect or Kerr nonlinearity* corresponds to the alteration in the refractive index (RI) of a substance in presence of an external electric field. It is linked with the real part (alternatively known as the refraction part) of third-order optical susceptibility [$\chi^{\left(3\right)}$] and possesses anchoring role in nonlinear optics owing to its importance in optical communications, quantum optics and optical devices [38], [39], [40]. In addition to this, nonlinear Kerr materials are promising candidates to be used in designing optical quantum computer [40], [41]. These applications require a large nonlinear Kerr effect [40] which can be achieved by exploiting the high nonlinearity of QD [42]. *DC-Kerr effect (DCKE)* (also known as *quadratic electro-optic effect; QEOE*) deems importance in view of realizing photoemission and detection applications of QDs [43]. Tuning and maximization of the magnitude and resonance wavelength of DCKE can be carried out by structural change of QD [43]. Amplified DCKE with depleted linear and nonlinear absorption are highly appreciated in varieties of quantum appliances as they make studies of nonlinear optics quite feasible at low light power [38], [39]. *Electro-absorption coefficient (EAC)* or *electro-absorption frequency-dependent susceptibility* is another notable third-order nonlinear optical (NLO) quantity to explore the photoemission and detection experiments of QDs [43]. Above facts have inspired lots of relevant works that deal with DCKE [38], [39], [40], [41], [42], [43], [44], [45], [46], [47], [48], [49], [50], [51] and EAC [43], [44], [45], [46], [48], [49], [50] in LDSS.

In case of Kerr-type nonlinearities the RI and absorption coefficient (AC) are given by $n=n_{0}+n_{2}I$ and $\alpha =\alpha_{0}+\alpha_{2}I$ where, *n*, $n_{0}$, $n_{2}$, *α*, $\alpha_{0}$, $\alpha_{2}$ and *I* are total RI, linear RI, nonlinear RI, total AC, linear AC, nonlinear AC and intensity of electromagnetic wave, respectively. For systems where absorption is insignificant ($\alpha_{0}∼0$), $n_{2}$ and $\alpha_{2}$ are found to be proportional to real and imaginary components of the complex quantity $\chi^{\left(3\right)}$, respectively. If the system is exposed to a single, monochromatic, linearly polarized field, the intensity of the field can be represented by $I=2\epsilon_{0}n_{0}c|F{|}^{2}$; where *c*, $\epsilon_{0}$, and |F| are the speed of light, electrical permittivity of free space ($=8.85\times 10^{-12}\;\text{F}/\text{m}$) and amplitude of the electric field, respectively. A notable change takes place in absorbing medium where the real and imaginary parts of first and third-order susceptibilities interplay between them and accordingly modulate $n_{2}$ and $\alpha_{2}$. In consequence, the expressions of $n_{2}$ and $\alpha_{2}$ are markedly altered (from that of non-absorbing one) and the aforesaid proportionality gets completely lost. It, therefore, appears important to measure the deviation in the magnitudes of $n_{2}$ and $\alpha_{2}$ in absorbing medium with respect to the non-absorbing medium. Such study helps us estimate the necessary corrections/rectifications needed to the magnitudes of $n_{2}$ and $\alpha_{2}$ in absorbing medium and the corrections are linked with the ratios of linear AC and linear RI and that of imaginary and real parts of $\chi^{\left(3\right)}$. Quantitatively, it is done by computing the *correction factor (CF)* for $n_{2}$ and $\alpha_{2}$ related to Kerr nonlinearity following a journey from *non-absorbing* to *absorbing media*. Regulated doping of impurity to LDSS can tune the magnitudes of $n_{0}$, $n_{2}$, $\alpha_{0}$, $\alpha_{2}$ and $\chi^{\left(3\right)}$, which, in turn, adjusts the two ratios as mentioned above. Such prudent manipulation of above quantities are conducive for generation of controlled nonlinear absorption effects and refractive nonlinearities [52].

Presence of *noise* influences the output of LDSS-based devices. Noise can enter the system through some external ‘modes’ or ‘pathways’. Two such pathways are usually called *additive* and *multiplicative* based on how system coordinates get coupled with noise. The physical properties of the system are modified as a result of ingression of noise and the extent of modification unveils conspicuous dependence on the particular mode. Therefore, it becomes obvious that the exploration of the noise effects on physical properties of LDSS is extremely important.

In the current manuscript we inspect how the *interplay between noise and binding energy (BE)* modulates the *DCKE, EAC and CF* of 2-d *GaAs* QD. Monitoring BE of LDSS deems importance as any change in BE heavily affects the physical properties of LDSS and therefore the designing of useful optoelectronic devices. The x−y confinement is described by harmonic oscillator potential and the *z*-confinement is offered by a perpendicular magnetic field. In addition, an electric field of strength *F* is applied to the system in *x* and *y*-directions. The QD contains *Gaussian impurity* as dopant and at the same time is exposed to *Gaussian white noise* applied via *additive* and *multiplicative* pathways (modes). The inspection illuminates how the interplay between BE and noise governs the aforesaid quantities with sufficient emphasis on the role performed by the noise mode.

## 2 Method

The Hamiltonian ($H_{0}$) of the system may be written as:

(1) $$H_{0}=H_{0}^{\prime }+V_{imp}+|e|F(x+y)+V_{noise}.$$

$H_{0}^{\prime }$ is the dopant-free Hamiltonian and *e* is the electronic charge. Use of effective mass approximation gives

(2) $$H_{0}^{\prime }=\frac{1}{2m^{⁎}}{\left[-i\hbar \nabla +\frac{e}{c}\text{A}\right]}^{2}+\frac{1}{2}m^{⁎}\omega_{0}^{2}(x^{2}+y^{2}).$$

$m^{⁎}$ and $\omega_{0}$ denote the effective mass of the electron and the harmonic confinement frequency, respectively. **A** is the vector potential given by A=(By,0,0), where *B* is the strength of the magnetic field. $H_{0}^{\prime }$ can also be written as

(3) $$H_{0}^{\prime }=-\frac{\hbar^{2}}{2m^{⁎}}\left(\frac{\partial^{2}}{\partial x^{2}}+\frac{\partial^{2}}{\partial y^{2}}\right)+\frac{1}{2}m^{⁎}\omega_{0}^{2}x^{2}+\frac{1}{2}m^{⁎}\Omega^{2}y^{2}-i\hbar \omega_{c}y\frac{\partial }{\partial x}.$$

$\Omega (=\sqrt{\omega_{0}^{2}+\omega_{c}^{2}})$ and $\omega_{c}(=\frac{eB}{m^{⁎}c})$ being the effective confinement frequency in the *y*-direction and the cyclotron frequency, respectively. $V_{imp}$ denotes the impurity potential and reads $V_{imp}=V_{0}\;e^{-\gamma \left[{\left(x-x_{0}\right)}^{2}+{\left(y-y_{0}\right)}^{2}\right]}$. Here $\left(x_{0},y_{0}\right)$, $V_{0}$ and $\gamma^{-1/2}$ stand for the dopant site (coordinate), dopant potential strength, and the spatial spread over which the influence of impurity persists, respectively.

$V_{noise}$ of eqn. (1) takes care of the contribution of noise to the Hamiltonian. In the present work Gaussian white noise has been exploited having features like *zero average* and *spatial δ-correlation*. Moreover, introduction of noise to the system is carried out in two different routes (called additive and multiplicative) which actually guide the size of system-noise interplay. Noise involves a spatially *δ*-correlated function [f(x,y)] which assumes a Gaussian distribution (produced by Box-Muller algorithm) having strength *ζ* and is described by the set of conditions

(4) 〈f(x,y)〉=0,

the zero average condition, and

(5) $$\langle f(x,y)f(x^{\prime },y^{\prime })\rangle =2\zeta \delta \left[(x,y)-(x^{\prime },y^{\prime })\right],$$

the spatial *δ*-correlation condition. The additive and multiplicative pathways of introduction of noise can be written as

(6) $$V_{noise}=\lambda_{1}f(x,y),$$

for additive pathway and

(7) $$V_{noise}=\lambda_{2}f(x,y)(x+y),$$

for the multiplicative pathway. $\lambda_{1}$ and $\lambda_{2}$ are two arbitrary parameters in case of additive and multiplicative noise, respectively. In reality, there exist a variety of physical situations in which external noise can be realized and bears interest. In these situations one deals with system which experiences fluctuations which are not *self-originating*. These fluctuations can be due to a fluctuating environment or can be consequence of an externally applied random force. Whereas additive noise does not interfere with the system coordinate the multiplicative analogue depends on the instantaneous value of the variables of the system. It does not scale with system size and is not necessarily small [53], [54]. We can regard the external noise as an external field which drives the system [54]. Experimentally, external noise can be generated by using a function generator (Hewlett-Packard 33120A) and its characteristics, viz. Gaussian distribution and zero mean can be maintained [55]. The external noise could be introduced multiplicatively using a circuit that enables to drive the nonlinear element by using the voltage from an external source [56].

Now, the construction of Hamiltonian matrix ($H_{0}$) [cf. eqn. (1)] has been carried out using the direct product basis of the harmonic oscillator eigenstates. The matrix elements corresponding to all the four components of eqn. (1) have been derived using the basis function mentioned above. It is followed by diagonalization of $H_{0}$ to compute the energy levels and the eigenstates of the system. The routine convergence test has been done during diagonalization.

Using the method of compact density matrix, within the framework of second-order perturbation theory, we consider the optical mixing of two incident light beams of frequencies $\nu_{1}$ and $\nu_{2}$. The corresponding third-order nonlinear optical susceptibility can then be written as [43]

(8) $$\chi^{\left(3\right)}(-2\nu_{1}+\nu_{2};\nu_{1},\nu_{1},-\nu_{2})=\frac{-2ie^{4}\sigma_{s}M_{ij}^{4}}{\varepsilon_{0}\hbar^{3}\left[i(\omega_{ij}-2\nu_{1}+\nu_{2})+\Gamma \right].\left[i(\nu_{2}-\nu_{1})+\Gamma \right]}\times \left[\frac{1}{i(\omega_{ij}-\nu_{1})+\Gamma }+\frac{1}{i(\nu_{2}-\omega_{ij})+\Gamma }\right],$$

where $\sigma_{s}$, $\varepsilon_{0}$, $M_{ij}=e\langle \psi_{i}|\overset{ˆ}{x}+\overset{ˆ}{y}|\psi_{j}\rangle$, $\psi_{i}(\psi_{j})$, $\omega_{ij}=(E_{i}-E_{j})/\hbar$ and $\Gamma =1/T_{2}$ stand for the carrier density, the vacuum dielectric constant, the dipole moment matrix elements, the eigenstates, the transition frequency and the relaxation rate with relaxation time $T_{2}$, respectively. During calculation, we have considered $\nu_{1}=0$ and $\nu_{2}=-\nu$ for convenience. The real and complex parts of $\chi^{\left(3\right)}$ are known as the DCKE and EAC, respectively, and reads [43]:

(9) $$\chi_{DCKE}^{\left(3\right)}(\nu )=Re\left[\chi^{\left(3\right)}(-\nu ,0,0,\nu )\right]$$

and

(10) $$\chi_{EAC}^{\left(3\right)}(\nu )=Im\left[\chi^{\left(3\right)}(-\nu ,0,0,\nu )\right].$$

The CF relevant to nonlinear RI in absorbing and non-absorbing media is given by [52]:

(11) $$\frac{n_{2}}{n_{2}(k_{0}=0)}={\left(1+\frac{k_{0}^{2}}{{n_{0}}^{2}}\right)}^{-1}\left[1+\frac{k_{0}}{n_{0}}\frac{\chi_{I}^{\left(3\right)}}{\chi_{R}^{\left(3\right)}}\right],$$

where $k_{0}=\frac{\lambda \alpha_{0}}{4\pi }$, *λ* is the wavelength of incident radiation, $\chi_{I}^{\left(3\right)}$ and $\chi_{R}^{\left(3\right)}$ are the complex and real parts of $\chi^{\left(3\right)}$, respectively. Analogously, the CF corresponding to nonlinear AC in absorbing and non-absorbing media is given by [52]:

(12) $$\frac{\alpha_{2}}{\alpha_{2}(k_{0}=0)}={\left(1+\frac{k_{0}^{2}}{n_{0}^{2}}\right)}^{-1}\left[1-\frac{k_{0}}{n_{0}}\frac{{\chi_{R}}^{\left(3\right)}}{\chi_{I}^{\left(3\right)}}\right].$$

The ground state binding energy $E_{B}$ can be written as

(13) $$E_{B}=E_{0}-E,$$

where *E* and $E_{0}$ are the ground state energies with and without impurity, respectively. In order to calculate the BE we have varied the impurity potential $V_{0}$ over a range (keeping all other parameters fixed). Thus, for different values of $V_{0}$ we get different BE values. Now, since a change of $V_{0}$ changes the Hamiltonian matrix $H_{0}$, the impurity BE enters as a dependent variable for the optical coefficients [cf. eqns. (8)–(12)].

## 3 Results and discussion

We have used ε=12.4 and $m^{⁎}=0.067m_{0}$ ($m_{0}$ is the mass of electron in vacuum). Moreover, during the study a few relevant parameters assume following fixed values: $\hbar \omega_{0}=250.0\;\text{meV}$ [57], [58], [59], B=20.0T [60], [61], F=100kV/cm [43], [59], $r_{0}=0.0\;\text{nm}$ and $\zeta =1.0\times 10^{-4}$, where *ζ* is the noise strength.

### 3.1 General aspects

**Figure 1:**
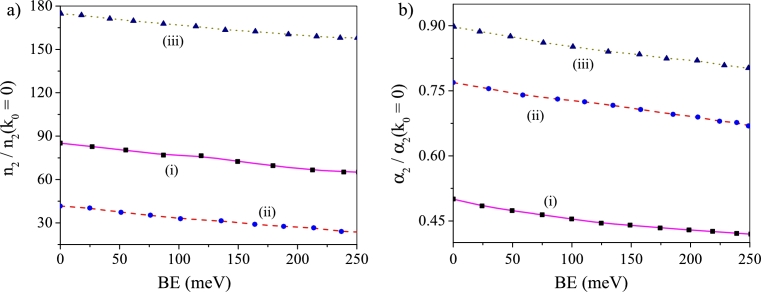
Plots of (a) $\frac{n_{2}}{n_{2}(k_{0}=0)}$ vs BE, and (b) $\frac{\alpha_{2}}{\alpha_{2}(k_{0}=0)}$ vs BE: In these plots (i) absence of noise, (ii) additive noise operates and (iii) multiplicative noise operates.

Figs. 1(a–b) display the plots of CF for RI and AC, respectively, with variation of BE. In both the plots (i), (ii) and (iii) indicate absence of noise, application of additive noise and application of multiplicative noise, respectively. CF for RI [Fig. 1a] and CF for AC [Fig. 1b] divulge similar behavior against a change of BE both with and without noise. For both RI and AC, regardless of applied noise and its pathway of introduction, CF displays steadfast fall as BE of the system increases.

**Figure 2:**
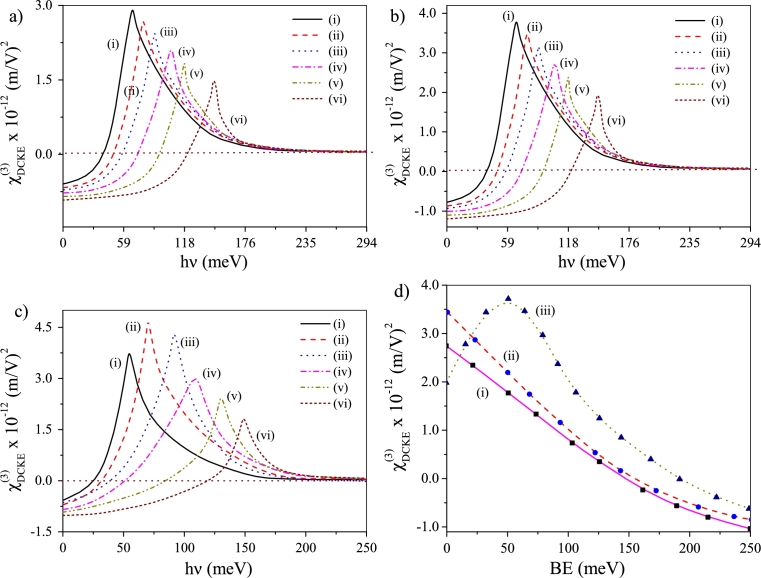
Plots of $\chi_{DCKE}^{\left(3\right)}$ vs *hν* for six different values of BE: (a) without noise, (b) additive noise operates and (c) multiplicative noise operates. The BE values are: (i) 0.0 meV, (ii) 50 meV, (iii) 100 meV, (iv) 150 meV, (v) 200 meV and (vi) 250 meV, (d) plots of $\chi_{DCKE}^{\left(3\right)}$ vs BE at *hν* = 70 meV where (i) noise-free, (ii) under additive noise and (iii) under multiplicative noise.

We now monitor the DCKE profiles as a function of oscillation frequency (*ν*) for six different values (given in the figure caption) of BE without noise effect [Fig. 2a] and when additive [Fig. 2b] and multiplicative [Fig. 2c] noise operate. Qualitatively similar features have been observed in the DCKE plots without noise and under additive noise. In both these cases we find regular drop of peak altitude and *blue-shift* of peaks as BE increases [44], [45], [46], [47], [48], [49], [50], [51]. Both of the above changes signify augmentation in the energy separation between the eigenstates and fall of dipole matrix element. Physically, the aforesaid changes in the energy separation and matrix element can be attributed to the enhanced confinement of the system because of increase in BE which shrinks the spatial spread of the wave function. Presence of multiplicative noise brings some departure in the DCKE profile from previous two situations. We now observe that the peak height undergoes *maximization* at BE∼50meV reflecting maximum overlap between the eigenfunctions around the above BE value induced by multiplicative noise; notwithstanding the spatial arrest imparted on the system. However, the erstwhile observed *blue-shift* of DCKE peaks with increase in BE is again observed. A better view of noise-BE interplay in the present context becomes available with the plot of DCKE against BE at hν=70.0meV [Fig. 2d]. The plot corroborates the previous observations revealed through monotonous drop in DCKE without noise [Fig. 2d(i)] and with additive noise [Fig. 2d(ii)], as BE increases. And with multiplicative noise, as expected, DCKE shows maximization at BE∼50meV [Fig. 2d(iii)].

**Figure 3:**
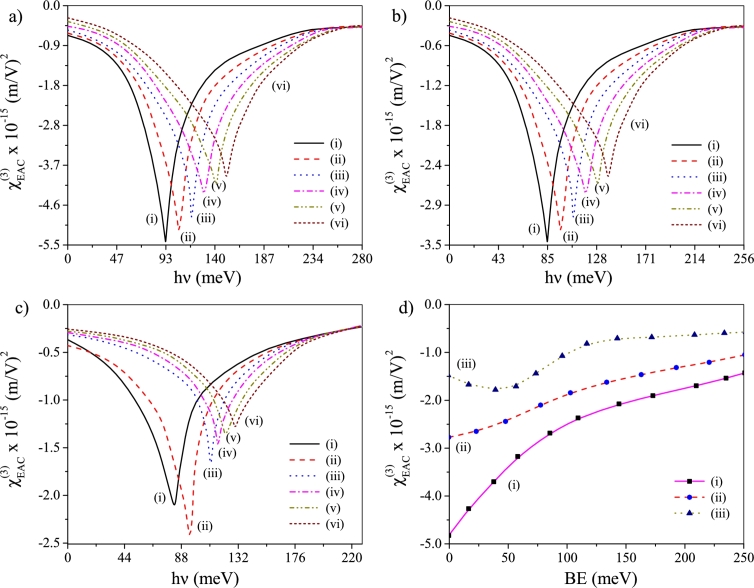
Plots of $\chi_{EAC}^{\left(3\right)}$ vs *hν* for six different values of BE: (a) without noise, (b) additive noise operates and (c) multiplicative noise operates. The BE values are: (i) 0.0 meV, (ii) 50 meV, (iii) 100 meV, (iv) 150 meV, (v) 200 meV and (vi) 250 meV, (d) plots of $\chi_{EAC}^{\left(3\right)}$ vs BE at *hν* = 90 meV where (i) noise-free, (ii) under additive noise and (iii) under multiplicative noise.

We now focus on exploring the influence of noise-BE interplay on EAC. EAC profiles are now plotted as a function of oscillation frequency (*ν*) for previously used values (given in the figure caption) of BE without noise effect [Fig. 3a] and when additive [Fig. 3b] and multiplicative [Fig. 3c] noise operate. Here also EAC profiles show similar characteristics without noise and with additive noise. In both these cases EAC peaks undergo *blue-shift* and their height gradually decreases as BE increases [43], [44], [45], [46], [47], [48], [49]. The observations can be realized on the basis of enhanced energy gap among the eigenstates and reduced magnitude of dipole matrix element. The said increase and the said reduction could have their origin in the spatial quenching of wave function as BE increases. Presence of multiplicative noise causes an alteration in the feature of EAC profile observed through the occurrence of *maximization* of the peak altitude at B∼50meV. However, previously envisaged *blue-shift* of EAC peaks as BE increases is again envisaged. The observation reflects that introduction of multiplicative noise causes maximization of mixing between the eigenstates around BE∼50meV superseding the spatial confinement imposed on the system. Fig. 3d plots EAC against BE itself at a given hν=90.0meV to further consolidate the above findings without noise [Fig. 3d(i)], with additive noise [Fig. 3d(ii)] and in presence of multiplicative noise [Fig. 3d(iii)], respectively. As expected, the plot depicts steady drop in EAC with progressive increase in BE in absence of noise and under additive noise, whereas EAC maximizes around BE∼50meV under the supervision of multiplicative noise.

Present investigation is believed to offer some *new physics*. It is ‘new’ since it is absolutely ‘rare’ to find any work that explores ‘noise’ effect on the important optical quantities like CF linked with Kerr nonlinearity, DCKE and EAC of doped LDSS. The LDSS is doped with impurity and therefore variation of impurity parameters obviously affects its BE. The situation assumes further importance with the incorporation of noise as now noise starts to interplay with the BE. Thus, the effective BE of the system becomes something different in presence of noise from that under noise-free condition. Moreover, the features of the noise mode (additive/multiplicative) also affect the effective BE mentioned above. All these things invariably influence the above optical properties in diverse ways that may have important practical relevance. In absence of noise we could not expect such diversities in the above optical properties and we stress to mention that herein lies the ‘new physics’. Already we have discussed in detail the findings of our study which amply reveal the important outcomes of noise-BE interplay/‘new physics’. Thus, now, we are not discussing it again for the brevity of the manuscript.

## 4 Conclusion

The influence of noise-BE interplay on CF linked with Kerr nonlinearity, DCKE and EAC of doped *GaAs* QD has been rigorously explored. The noise-BE interplay does not qualitatively affect the CF as under all situations CF declines with enhancement of BE. The said interplay, however, displays some noticeable role in case of DCKE and EAC. For these two properties the mode of introduction of noise modulates the said interplay. Both of these properties exhibit steady drop and blue-shift as BE increases; without noise and when additive noise operates. Presence of multiplicative noise, though does not affect the general trend of peak shift, but causes maximization of peak height at BE∼50meV. A switch from additive to multiplicative mode simply causes a change in the manner noise couples with the system coordinates. Naturally, the noise-BE interaction also depends on the aforesaid mode giving rise to distinct features in DCKE and EAC profiles.

## Declarations

### Author contribution statement

Anuja Ghosh, Sk. Md. Arif, Manas Ghosh: Conceived and designed the experiments; Performed the experiments; Analyzed and interpreted the data; Contributed reagents, materials, analysis tools or data; Wrote the paper.

### Funding statement

This research did not receive any specific grant from funding agencies in the public, commercial or not-for-profit sectors.

### Competing interest statement

The authors declare no conflict of interest.

### Additional information

No additional information is available for this paper.
